# Aged Mice Have Enhanced Endocortical Response and Normal Periosteal Response Compared With Young-Adult Mice Following 1 Week of Axial Tibial Compression

**DOI:** 10.1002/jbmr.96

**Published:** 2010-03-26

**Authors:** Michael D Brodt, Matthew J Silva

**Affiliations:** 1 Department of Orthopaedic Surgery, Washington University St. Louis, MO, USA; 2 Department of Biomedical Engineering, Washington University St. Louis, MO, USA

**Keywords:** bone formation, mouse, mechanoresponse, aging, tibial compression

## Abstract

With aging, the skeleton may lose its ability to respond to positive mechanical stimuli. We hypothesized that aged mice are less responsive to loading than young-adult mice. We subjected aged (22 months) and young-adult (7 months) BALB/c male mice to daily bouts of axial tibial compression for 1 week and evaluated cortical and trabecular responses using micro–computed tomography (µCT) and dynamic histomorphometry. The right legs of 95 mice were loaded for 60 rest-inserted cycles per day to 8, 10, or 12 N peak force (generating mid-diaphyseal strains of 900 to 1900 µɛ endocortically and 1400 to 3100 µɛ periosteally). At the mid-diaphysis, mice from both age groups showed a strong anabolic response on the endocortex (Ec) and periosteum (Ps) [Ec.MS/BS and Ps.MS/BS: loaded (right) versus control (left), *p* < .05]. Generally, bone formation increased with increasing peak force. At the endocortical surface, contrary to our hypothesis, aged mice had a significantly greater response to loading than young-adult mice (Ec.MS/BS and Ec.BFR/BS: 22 months versus 7 months, *p* < .001). Responses at the periosteal surface did not differ between age groups (*p* > .05). The loading-induced increase in bone formation resulted in increased cortical area in both age groups (loaded versus control, *p* < .05). In contrast to the strong cortical response, loading only weakly stimulated trabecular bone formation. Serial (in vivo) µCT examinations at the proximal metaphysis revealed that loading caused a loss of trabecular bone in 7-month-old mice, whereas it appeared to prevent bone loss in 22-month-old mice. In summary, 1 week of daily tibial compression stimulated a robust endocortical and periosteal bone-formation response at the mid-diaphysis in both young-adult and aged male BALB/c mice. We conclude that aging does not limit the short-term anabolic response of cortical bone to mechanical stimulation in our animal model. © 2010 American Society for Bone and Mineral Research.

## Introduction

Rates of bone formation diminish with aging in humans and mice.(1–4) One reason for this decline in bone formation may be reduced responsiveness to the mechanical stimuli produced by physical activity. However, our understanding of aging and skeletal responses to loading is incomplete. Animal studies, which offer the benefit of controlled loading exposure, have not led to a consensus. Exercise studies that subjected young and aged rodents to running or jumping protocols have reported either reduced responsiveness in aged animals,(5,6) no difference between ages,(7–9) or enhanced responsiveness in aged animals.(10,11) Studies that used direct skeletal overloading (ie, ulnar compression or tibial bending) have been more consistent, with reports of reduced responsiveness in aged turkeys,(12) rats,(13) and mice(14) compared with younger animals. The basis for these findings is unclear because at least one in vitro study showed that there was no cell autonomous decline in mechanoresponsiveness with aging.(15)

Prior studies of direct skeletal overloading in aged animals used approaches that were either invasive (eg, avian ulnar compression(12)) or loaded bones in a nonphysiologic direction (eg, tibial four-point bending(13) and tibial cantilever bending(14)). Our objective was to extend these previous studies by using a noninvasive method that loads the tibia in a physiologic direction. In addition, we sought to evaluate loading responses at both cortical and trabecular sites. Therefore, we subjected aged and young-adult mice to daily bouts of axial tibial compression for 1 week and evaluated cortical and trabecular responses using micro–computed tomography (µCT) and dynamic histomorphometry. We hypothesized that aged mice have reduced responsiveness to loading compared with young-adult mice.

## Methods

### Overview of in vivo loading

The right tibias of adult male BALB/c mice (National Institutes of Aging/Harlan, Bethesda, MD, USA) were subjected to noninvasive axial compression based on a method described by others.(16,17) Mice were aged either 7 to 8 or 21 to 22 months at the start of loading. (We refer to these groups as “7 months” or “22 months” corresponding to the majority age.) BALB/c mice were selected because they represent an inbred strain with intermediate bone mass [between the extremes of C57Bl/6 (low) and C3H/He (high)](18) and because they exhibit age-related skeletal changes that mimic those seen in humans.(19) For loading, the right legs were positioned vertically (foot up, knee down) in a fixture attached to a servohydraulic loading machine (Instron Dynamite 8800, Norwood, MA, USA). Compressive loading was applied, through contacts at the knee and foot, under force control using a rest-inserted waveform(14) (triangle waveform to peak force at 48 N/s load and unload, followed by a 10-second rest interval). Left legs were not loaded and served as contralateral controls. A recent study using a similar loading protocol found no effect of loading on contralateral bones.(20) This study was approved by our institutional Animal Studies Committee and was conducted in accordance with the Public Health Service Policy on Humane Care and Use of Laboratory Animals.

### Force-strain analysis

**Fig. 1:**
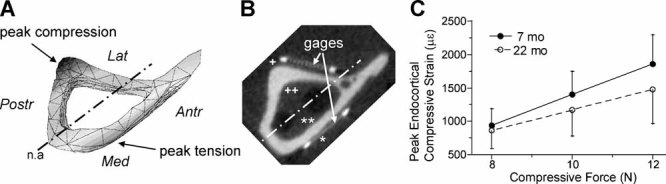
(*A*) Transverse tibial cross section from finite-element model^21^ showing distribution of axial strain (strain component along the tibial axis, normal to the page) during axial compression. (*B*) µCT image of tibia from 22-month-old BALB/c mouse showing location of gauges for experimental strain measurements. Strain data were scaled based on beam theory to estimate strain values at sites of peak tension and compression on both endocortical and periosteal surfaces. (*C*) Linear force-strain relationship for strain at the peak endocortical site. Strain magnitude (mean ± SD) did not differ significantly between 7- and 22-month-old tibias. In vivo loading was performed at 8, 10, and 12 N force levels (+ site of peak periosteal compressive strain; ++ site of peak endocortical compressive strain; * site of peak periosteal tensile strain; ** site of peak endocortical tensile strain).

Prior to in vivo loading, we determined force-strain relationships for tibial compression in mice at ages 7 (*n* = 5) and 22 (*n* = 4) months using a combination of strain-gauge measurements, µCT imaging, and engineering beam theory. Our goal was to determine force values that produced peak endocortical strains in the 800- to 2000-µɛ range. Owing to the curvature of the mouse tibia, axial compression generates combined compression-bending at the mid-diaphysis. Previous analysis indicated that the site of peak axial strain is near the mid-diaphysis, approximately 5 mm proximal to the distal tibiofibular junction (TFJ).(21) In the transverse plane, peak compressive strain occurs near the posterolateral apex of the tibia, whereas peak tensile strain is on the opposite anteromedial face (Fig. 1*A*). Two single-element strain gauges (EA06-015LA-120, Vishay Measurements Group, Raleigh, NC, USA) were attached as close as possible to the sites of peak strain. Cyclic compressive loading was applied to peak-force magnitudes of 4 to 18 N (2-N increments), and peak strains were recorded. Force versus strain regression lines were determined for each tibia (*r*^2^ > 0.85). Tibias then were scanned at the gauge site by µCT (µCT 40, Scanco Medical, Brüttisellen, Switzerland). From cross-sectional images, the bone centroid, area, and moment of inertia about the neutral axis were determined, as well as distances from the centroid to the gauge locations (*y*_gauge_) and to the predicted sites of peak endocortical and periosteal strain (y*) (Fig. 1*B*). Based on a beam-theory approach previously validated,(22) we then extrapolated the strain-gauge data to estimate strain values at the sites of maximal endocortical and periosteal strain using the relation ɛ* = ɛ_gauge_(y*/*y*_gauge_), where * denotes the site of interest (eg, peak endocortical strain). Compressive and tensile values were based on the gauges on the compressive and tensile sides, respectively. From this analysis, we selected force levels of 8, 10, and 12 N for in vivo loading (Table 1; Fig. 1*C*). Moments of inertia were not significantly different between age groups (7 months: 0.077 ± 0.016 mm^4^; 22 months: 0.084 ± 0.006 mm^4^; *p* = .21), and thus values of strain for a given force were similar for 7- and 22-month tibias (*p* > .05).

**Table 1:** Mid-diaphyseal Compressive Strains (Microstrain, Mean ± SD) at Periosteal Gauge Site (Measured) and at Sites Where Peak Endocortical and Periosteal Strains Are Predicted for 7- and 22-Month-Old Tibias

	Gauge site		Peak endocortical		Peak periosteal	
			
Force (N)	7 Months	22 Months	7 Months	22 Months	7 Months	22 Months
−8	−840 ± 300	−720 ± 210	−940 ± 250	−860 ± 270	−1550 ± 380	−1360 ± 420
−10	−1250 ± 420	−980 ± 300	−1400 ± 350	−1170 ± 390	−2320 ± 500	−1860 ± 600
−12	−1660 ± 540	−1240 ± 380	−1860 ± 440	−1480 ± 510	−3090 ± 640	−2350 ± 790

### Study 1: In vivo loading with postmortem assessment

We performed in vivo loading on 71 mice (7 months: *n* = 36; 22 months: *n* = 35). Under anesthesia, the right legs were loaded to a peak force of 8, 10, or 12 N for 60 cycles/day for 5 days (M–F); left legs were not loaded (control). To label new bone formation, mice were injected with fluorochromes on days 4 (calcein green, 7.5 mg/kg i.p.; Sigma, Saint Louis, MO, USA) and 9 (alizarin complexone, 30 mg/kg i.p.; Sigma) and killed on day 11 by CO_2_ asphyxiation. Seven mice died prior to the end of the study: Two died from anesthesia overdose, one was euthanized to relieve distress, and four were euthanized after sustaining a tibial fracture during loading. For the remaining 64 mice, bilateral tibias were harvested immediately postmortem, fixed for 24 hours in 4% paraformaldehyde, and stored in 70% ethanol.

Body weights differed slightly between age groups at the start of the study (7 months: 31.3 ± 2.0 g; 22 months: 28.4 ± 3.7 g; *p* < .001) and at the end of the experiment (7 months: 30.1 ± 1.8 g; 22 months: 26.4 ± 3.7 g; *p* < .001). Mice lost an average of 4% (7 months) and 7% (22 months) of body weight during the experiment. Importantly, body weights did not differ between force groups (*p* > .05) either at the start or the end of the study.

Tibias were scanned by µCT to assess trabecular bone morphology in the proximal metaphysis (55 kVp, 145 mA, standard resolution, 16.4-mm diameter, 16-µm voxel size, 300-ms integration time). The volume of interest was located just distal to the physis, spanning a height of 480 µm (30 slices) and containing all bone inside the cortical shell. Using the manufacturer's 3D analysis tools, we determined apparent volumetric bone mineral density (vBMD), bone volume fraction (BV/TV), trabecular thickness (Tb.Th), trabecular separation (Tb.Sp), and trabecular number (Tb.N) (based on the direct method of calculation(23)). A threshold of 30% of maximum grayscale value (300/1000) was used to segment bone from nonbone.

Indices of bone formation were analyzed using standard histomorphometry. Tibias were dehydrated and embedded in methyl methacrylate (Sigma).(24) For analysis of cortical bone, duplicate 100-µm-thick transverse sections were cut (Leica 1600SP, Wetzlar, Germany) from each tibia 5 mm proximal to the distal TFJ. Slides were mounted on glass, and two-color fluorescent images were obtained using a confocal microscope (LSM 510, Axiovert 200M, Plan-Neofluar 10 × /0.30 NA Objective, Carl Zeiss, Jena, Germany). Dynamic histomorphometric analysis was performed using commercial software (OSTEO II, Bioquant, Nashville, TN, USA). Results from the duplicate sections were averaged. We determined single- and double-labeled surface per bone surface (sLS/BS, dLS/BS), mineralizing surface (MS/BS), mineral apposition rate (MAR), and bone-formation rate (BFR/BS), as defined elsewhere.(25) For each section, we analyzed the entire endocortical (Ec) and periosteal (Ps) surfaces separately. *Single-labeled surface* was defined as surface labeled with red, green, or yellow (red and green overlaid without separation). *Double-labeled surface* was defined where both red and green labels were present with separation. MS/BS was calculated as 0.5 × sLS/BS + dLS/BS for all samples. If a sample had no double-labeled surface (dLS/BS = 0), it was coded as “no data” for MAR and BFR/BS based on a published recommendation.(26) We also determined static morphometric parameters: medullary area (Med.Ar), bone area (B.Ar), and cortical width (Ct.Wi). For analysis of trabecular bone, thick sections were cut in the frontal plane from the proximal tibias. Sections were imaged and analyzed as described earlier, with the region of interest (ROI) corresponding to the ROI for µCT analysis of the proximal tibial metaphysis.

### Study 2: In vivo loading with in vivo µCT

A follow-up study was performed in 24 mice to evaluate serial changes in trabecular bone. The proximal tibial metaphysis was scanned in vivo by µCT (VivaCT 40, Scanco Medical) 3 days prior to loading. Tibias were loaded for 5 days (M–F) using the same protocol as study 1 and rescanned 4 days after the end of loading (the time of the second fluorochrome label in study 1). (The cortical diaphysis also was scanned at both time points.) Because results of study 1 (see below) showed no effect of force level on trabecular parameters, we used a single force level for study 2. Mice aged 7 months (*n* = 12) were loaded to a peak force of 12 N on days 1 through 5. Mice aged 22 months (*n* = 12) were loaded to a peak force of 12 N on day 1 and 10 N on days 2 through 5; the force level was reduced because three 22-month-old mice sustained fractures on day 1. Another two 22-month-old mice died during the study from anesthesia overdose. After the second µCT scan, mice were killed, and bilateral tibias harvested. Tibias were decalcified and embedded in paraffin. Midsagittal sections (5 µm) were cut and stained for tartrate resistant acid phosphatase (TRACP)(27) to label osteoclasts. Trabecular osteoclast surface (Oc.S/BS) was determined in the region corresponding to the ROI for µCT of the proximal tibial metaphysis.

### Statistical analysis

Differences between loaded (R) and nonloaded (L) legs were assessed by paired *t* tests (Statview 5.0, SAS Institute, Cary, NC, USA). Effects of age and force level were assessed by two-way analysis of variance (ANOVA). ANOVA was performed on absolute data from the left and right legs as well as on relative (*r*) data (ie, right to left). When the overall ANOVA reached significance for force level, post hoc differences were evaluated using Fisher's protected least squares difference (PLSD) test. For study 2, changes with time were assessed using paired *t* tests (before versus after). Differences were defined as statically significant at *p* < .05.

## Results

### Study 1: Control tibias

Tibias from 22-month-old mice were osteopenic compared with those of 7-month-old mice. Force level had no significant effect on morphology of the left tibia, so data from the 8-, 10-, and 12-N groups were pooled. Medullary area was 32% greater in 22- versus 7-month-old tibias (*p* < .001), whereas cortical area was 18% less (*p* < .001), and cortical width was 23% less (*p* < .001). Trabecular BV/TV was 54% less in 22- versus 7-month-old tibias; Tb.N, Tb.Th, and vBMD were 6%, 10%, and 34% less (*p* < .01; Table 2).

**Table 2:** Cortical Bone Morphology at the Mid-diaphysis and Trabecular Bone Morphology and Density at the Proximal Tibial Metaphysis (Mean ± SD)

		7 Months		22 Months	
			
Site	Outcome	Control (*n* = 31–34)	Loaded (*n* = 32)	Control (*n* = 28–30)	Loaded (*n* = 28–29)
Cortical	Med.Ar (mm^2^)	0.391 ± 0.056	0.405 ± 0.054	0.517^b^ ± 0.065	0.492^b^ ± 0.101
	B.Ar (mm^2^)	0.723 ± 0.068	0.762^a^ ± 0.055	0.590^b^ ± 0.070	0.631^a^,^b^ ± 0.057
	Ct.Wi (mm)	0.196 ± 0.012	0.201 ± 0.016	0.151^b^ ± 0.016	0.161^a^,^b^ ± 0.013
Trabecular	BV/TV (mm^3^/mm^3^)	0.132 ± 0.032	0.098^a^ ± 0.018	0.061^b^ ± 0.019	0.067^b^ ± 0.023
	Tb.N (1/mm)	4.9 ± 0.3	4.8 ± 0.3	4.6^b^ ± 0.5	4.5^b^ ± 0.4
	Tb.Th (mm)	0.062 ± 0.005	0.059^a^ ± 0.005	0.056^b^ ± 0.006	0.060^a^ ± 0.006
	vBMD (mg HA/cm^3^)	230 ± 23	210^a^ ± 16	147^b^ ± 26	149^b^ ± 24

*Note: Force* groups are pooled because there is no effect of force level (*p* > .05 by two-way ANOVA).

a Loaded (right) different from control (nonloaded, left); *p* < .05, paired *t* test.

b Twenty-two-month-old group different from 7-month-old group; *p* < .05, ANOVA.

Analysis of cortical bone formation in nonloaded tibias revealed single-labeled surface in all bones, but a low incidence of double-labeled surface in both age groups. Only 5 of 59 bones had endocortical double label (Table 3), and only 5 of 59 had periosteal double label (Table 4). There was no effect of force level on bone-formation indices of nonloaded tibias, so data were pooled to compare ages. Endocortical sLS/BS and MS/BS were 30% greater in 22- versus 7-month-old tibias (*p* = .004). Periosteal indices did not differ between age groups.

**Table 3:** Endocortical Bone-Formation Indices From Mice Subjected to Axial Tibial Compression (Mean ± SD)

	7 Months						22 Months					
		
	8 N		10 N		12 N		8 N		10 N		12 N	
						
Outcome	Control (*n* = 11)	Loaded (*n* = 12)	Control (*n* = 13)	Loaded (*n* = 12)	Control (*n* = 7)	Loaded (*n* = 8)	Control (*n* =11)	Loaded (*n* = 10)	Control (*n* = 8)	Loaded (*n* = 9)	Control (*n* = 9)	Loaded (*n* = 9)
Ec.sLS/BS (%)	28.9 ± 9.7	29.0 ± 11.1	25.4 ± 8.0	31.8^a^ ± 9.2	29.2 ± 6.9	39.0 ± 23.6	34.8 ± 12.4	50.4^d^ ± 15.1	33.0 ± 12.8	48.8^a^,^d^ ± 13.2	39.3^d^ ± 7.7	46.2 ± 18.1
Ec.dLS/BS (%)	0.1 ± 0.3	0.9 ± 1.9	0.2 ± 0.6	1.0 ± 1.7	0.0 ± 0.0	4.5^a^,^b^,^c^ ± 4.2	0.2 ± 0.5	4.4^a^,^d^ ± 4.4	0.1 ± 0.2	13.9 a,b,d ± 11.1	0.0 ± 0.0	17.3^a^,^b^,^d^ ± 14.7
Ec.MS/BS (%)	14.2 ± 5.6	15.4 ± 5.9	12.9 ± 3.8	16.9^a^ ± 5.6	14.6 ± 3.4	24.0 a,b,c ±10.7	17.6 ± 6.2	29.6^a^,^d^ ± 9.2	16.6 ± 6.6	38.2^a^,^d^ ± 12.3	19.7^d^ ± 3.9	40.4^a^,^d^ ± 15.4
Ec.MAR (µm/day)	0.75 (*n* = 1)	1.29 ± 0.33 (*n* = 4)	1.63 (*n* = 1)	1.41 ± 0.64 (*n* = 5)	ND	1.42 ± 0.60 (*n* = 6)	1.13 (*n* = 2)	1.52 ± 0.36 (*n* = 8)	1.03 (*n* = 1)	1.71 ± 0.52 (*n* = 9)	ND	1.92 ± 0.52 (*n* = 9)
Ec.BFR/BS (µm/day)	0.13 (*n* = 1)	0.22 ± 0.03 (*n* = 4)	0.21 (*n* = 1)	0.30 ± 0.11 (*n* = 5)	ND	0.32 ± 0.21 (*n* = 6)	0.27 (*n* = 2)	0.49 ± 0.25 (*n* = 8)	0.26 (*n* = 1)	0.70^d^ ± 0.33 (*n* = 9)	ND	0.82^d^ ± 0.44 (*n* = 9)

a Loaded (right) different from control (nonloaded, left); *p* < .05, paired *t* test.

b Different from 8-N group of same age.

c Different from 10-N group of same age; *p* < .05, ANOVA.

d Different from 7-month-old group at same force level; *p* < .05, ANOVA.

ND = no detectable double label; owingue to the low incidence of double labels in control tibias, statistical comparisons of MAR and BFR between loaded and control bones were not performed.

**Table 4:** Periosteal Bone Formation Indices From Mice Subjected to Axial Tibial Compression (Mean ± SD)

	7 Months						22 Months					
		
	8 N		10 N		12 N		8 N		10 N		12 N	
						
Outcome	Control (*n* = 11)	Loaded (*n* = 12)	Control (*n* = 13)	Loaded (*n* = 12)	Control (*n* = 7)	Loaded (*n* = 8)	Control (*n* = 11)	Loaded (*n* = 10)	Control (*n* = 8)	Loaded (*n* = 9)	Control (*n* = 9)	Loaded (*n* = 9)
Ps.Sls/BS (%)	23.0 ± 15.6	41.5^a^ ± 11.6	20.3 ± 13.9	62.7^a^,^b^ ± 10.8	18.0 ± 8.5	53.2^a^,^b^ ± 12.7	20.4 ± 12.0	47.3^a^ ± 18.5	17.5 ± 7.0	57.1^a^ ± 11.7	20.1 ± 3.7	52.9^a^ ± 10.8
Ps.dLS/BS (%)	0.7 ± 1.6	4.1 ± 4.8	0.4 ± 1.2	6.7^a^ ± 10.2	0.0 ± 0.0	7.6^a^ ± 8.8	0.2 ± 0.8	1.4 ± 3.2	0.0 ± 0.0	3.8 ± 5.5	0.0 ± 0.0	8.0^a^,^b^ ± 6.3
Ps.MS/BS (%)	12.2 ± 8.9	24.8^a^ ± 9.4	10.5 ± 7.7	38.1^a^,^b^ ± 9.2	9.0 ± 4.3	34.2^a^ ± 12.2	10.5 ± 6.5	25.1^a^ ± 9.6	8.8 ± 3.5	32.3^a^ ± 7.4	10.0 ± 1.8	34.5^a^,^b^ ± 9.8
Ps.MAR (µm/day)	1.08 (*n* = 2)	1.46 ± 0.27 (*n* = 9)	1.14 (*n* = 2)	1.74 ± 0.45 (*n* = 11)	ND	2.48^b^,^c^ ± 0.96 (*n* = 6)	1.76 (*n* = 1)	1.29 (*n* = 2)	ND	2.37 ± 0.75 (*n* = 4)	ND	2.14 ± 0.40 (*n* = 9)
Ps.BFR/BS (µm/day)	0.27 (*n* = 2)	0.43 ± 0.13 (*n* = 9)	0.30 (*n* = 2)	0.72^b^ ± 0.33 (*n* = 11)	ND	0.96^b^ ± 0.34 (*n* = 6)	0.64 (*n* = 1)	0.37 (*n* = 2)	ND	0.82 ± 0.35 (*n* = 4)	ND	0.75 ± 0.28 (*n* = 9)

a Loaded (right) different from control (nonloaded, left); *p* < .05, paired *t* test.

b Different from 8-N group of same age.

c Different from 10-N group of same age; *p* < .05, ANOVA.

ND = no detectable double label; owing to the low incidence of double labels in control tibias, statistical comparisons of MAR and BFR between loaded and nonloaded bones were not performed.

### Study 1: Effects of loading on cortical bone

**Fig. 2:**
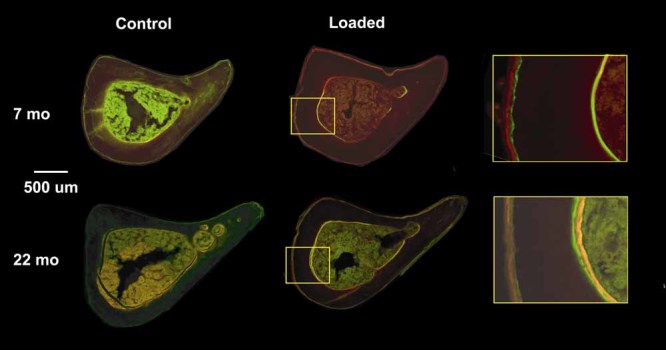
Fluorescent photomicrographs of mid-diaphyseal tibial sections from loaded and control tibias of 22- and 7-month-old mice. Samples were collected on day 11 following tibial compression on days 1 through 5 and fluorochrome labeling on days 5 (*green*) and 10 (*red*). An increase in endocortical and periosteal labeled surface is evident in loaded tibias of both age groups compared with controls. (Shown are tibias from the 10-N load group.)

**Fig. 3:**
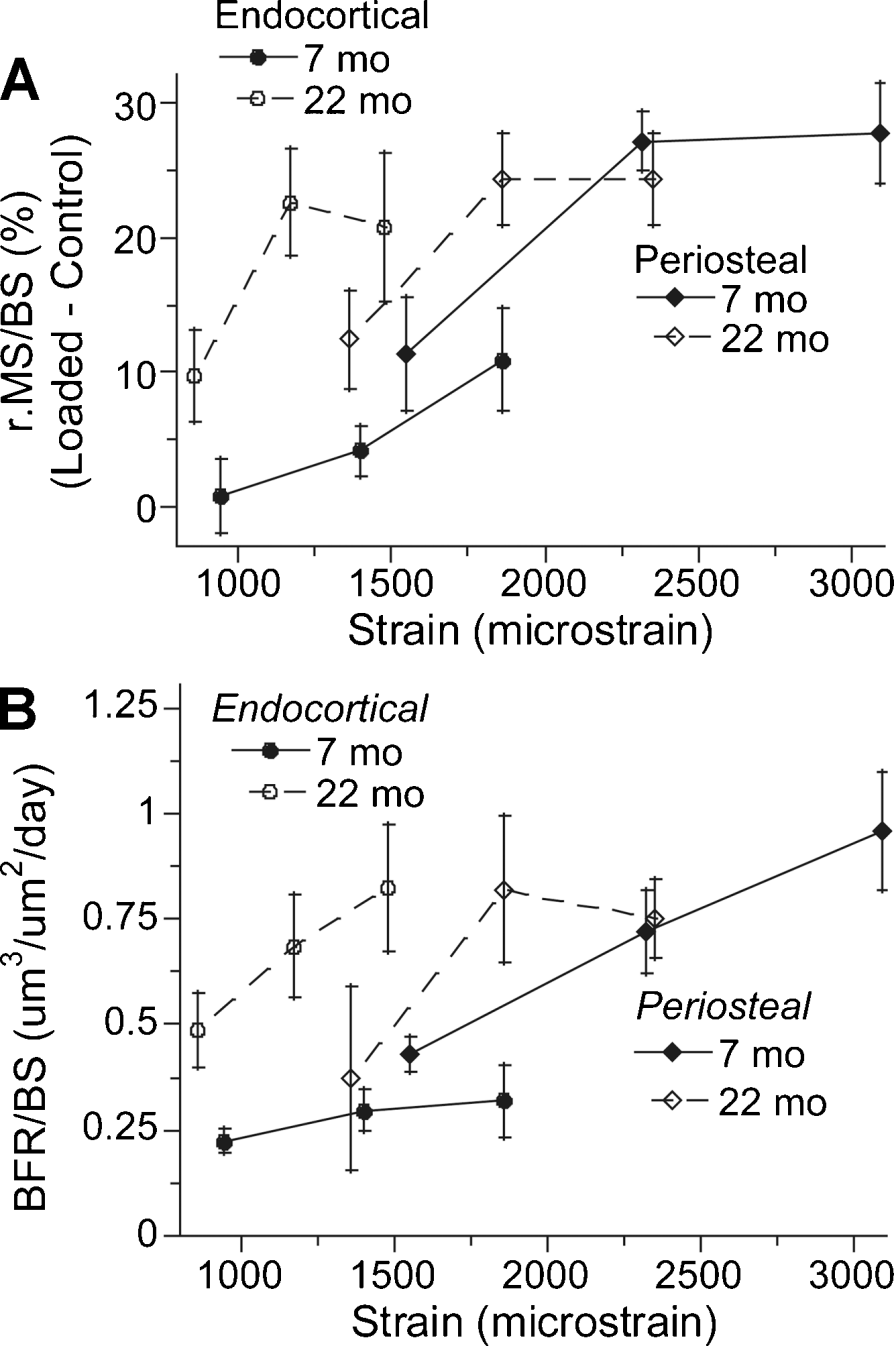
Cortical bone-formation indices from tibias of loaded mice versus estimated peak compressive strain magnitude (mean ± SE). (*A*) Relative mineralizing surface and (*B*) absolute bone-formation rate were significantly greater in 22- than 7-month-old mice at the *endocortical* surface (*p* < .001), whereas there was no difference between age groups at the *periosteal* surface. A dose response is evident, with greater bone formation with increasing strain magnitude. Interestingly, the endocortical and periosteal curves are roughly continuous for the 7-month-old groups, with overlap at the highest endocortical strain and lowest periosteal strain. In contrast, the curves for the 22-month-old groups do not appear to overlap; a maximal response on the endocortical surface occurs at a strain level (∼1500 µɛ) that causes a submaximal periosteal response. Thus it appears that the endocortical and periosteal surfaces have a similar mechanosensitivity in 7-month-old mice, whereas the endocortical surface of 22-month-old mice is surprisingly elevated compared with the periosteal surface.

Analysis of bone-formation indices at the cortical diaphysis (site of maximal strain) in loaded tibias revealed that contrary to our hypothesis, aged mice did not have diminished responsiveness to loading (Figs. 2 and 3). Bone formation occurred primarily as lamellar bone, with small amounts of woven bone evident in only six 22-month-old mice (two 10 N, four 12 N) and two 7-month-old mice (12 N). On the *endocortical* surface, in both 22- and 7-month-old mice, loaded tibias had greater Ec.dLS/BS and Ec.MS/BS values than control tibias (*p* < .01; Table 3), indicating a significant loading response. ANOVA indicated that loaded tibias from 22-month-old mice had greater Ec.sLS/BS, Ec.dLS/BS, Ec.MS/BS, and Ec.BFR/BS values than 7-month-old mice (*p* < .001). ANOVA of relative differences (loaded to control) also indicated that r.Ec.dLS/BS and r.Ec.MS/BS values were greater in 22- than in 7-month-old mice (*p* < .001). (Relative values for MAR and BFR/BS were not computed because of the lack of double-labeled bone on the control side.) On the *periosteal* surface, there also was a significant loading effect in both 22- and 7-month-old mice, with Ps.sLS/BS, Ps.dLS/BS, and Ps.MS/BS values significantly greater in loaded versus control tibias (*p* < .001; Table 4). ANOVA revealed no significant differences between 22- and 7-month-old mice in terms of either absolute or relative effects of loading, indicating a similar periosteal response for the two ages.

Each mouse leg was loaded at either 8, 10, or 12 N peak force, which produced average endocortical compressive strains at the tibial mid-diaphysis of 900, 1290, and 1670 µɛ, respectively (Table 1; Fig. 1*C*). Both age groups had evidence of a dose-response effect, especially on the periosteal surface, with a general finding of greater bone formation in the 10- and 12-N groups compared with the 8-N group. On the *endocortical* surface of loaded tibias, Ec.dLS/BS and Ec.MS/BS values depended on force level (*p* < .05 by two-way ANOVA), whereas on the *periosteal* surface, Ps.sLS/BS, Ps.MS/BS, Ps.MAR, and Ps.BFR/BS values depended on force level (*p* < .05). For example, for 7-month-old mice, loaded tibias in the 10- and 12-N groups had significantly greater Ps.sLS/BS and Ps.BFR/BS values than those in the 8-N group; for 22-month-old mice, loaded tibias in the 12-N group had significantly greater Ps.dLS/BS and Ps.MS/BS values than those in the 8-N group (Table 4).

The increase in bone-formation rates induced by mechanical loading resulted in increased cortical area and width in both age groups. In 7-month-old mice, cortical area was 5% greater in loaded tibias than in nonloaded controls (*p* = .020; Table 2). In 22-month-old mice, both cortical area and cortical width were 7% greater in loaded tibias than in nonloaded controls (*p* < .001).

### Study 1: Effects of loading on trabecular bone

**Fig. 4:**
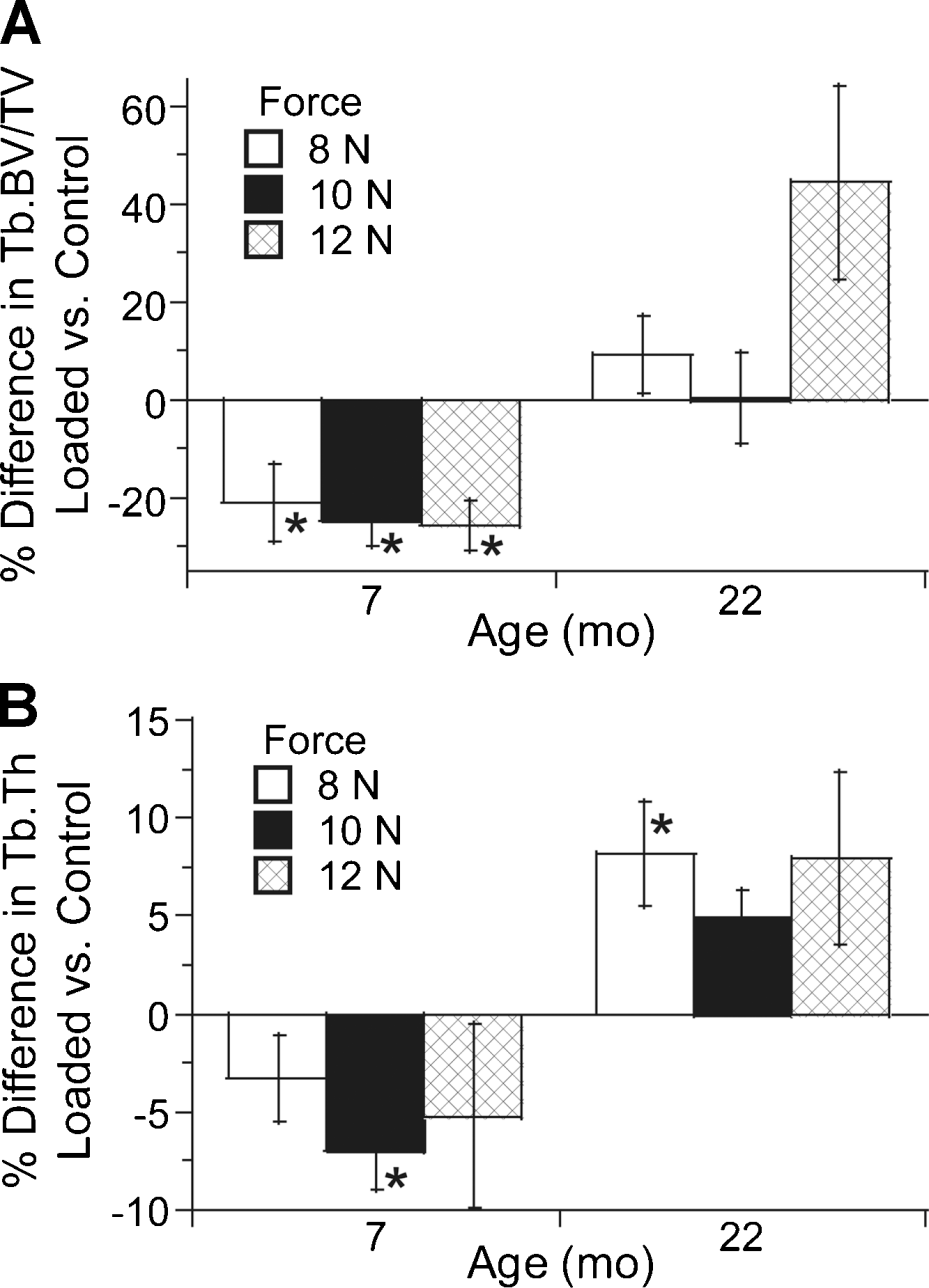
Differences in (*A*) trabecular bone volume fraction and (*B*) trabecular thickness between loaded and control tibias determined by postmortem µCT of the proximal tibia (study 1) (mean ± SE). In 7-month-old mice, BV/TV was approximately 25% less in loaded tibias than controls, whereas Tb.Th was 5% less (*p* < .01 by ANOVA). In 22-month-old mice, by contrast, BV/TV did not differ between loaded and control tibias (*p* = .55), whereas Tb.Th was approximately 7% greater in loaded tibias (*p* < .001). *Loaded different from control; *p* < .05, paired *t* test.

Trabecular bone formation in the proximal tibial metaphysis was modestly enhanced by loading, whereas trabecular structure was strongly negatively affected. In 7-month-old mice, loaded tibias in the 10- and 12-N groups had a significantly higher trabecular MS/BS compared with controls (Table 5). Similar trends were observed in 22-month-old mice but did not reach significance. Loading did not reliably induce mineral apposition because only 19 of 55 loaded tibias had nonzero trabecular double label. In terms of trabecular structure, BV/TV, Tb.Th, and vBMD values in 7-month-old mice were all unexpectedly less in loaded tibias than in control tibias (*p* < .05; Table 2; Fig. 4). In 22-month-old mice, by contrast, trabecular BV/TV and vBMD values did not differ between loaded and control tibias, whereas Tb.Th was greater in loaded than in control tibias (*p* < .001). There were no significant effects of force level on trabecular µCT parameters for either age group. Thus loading appears to have caused loss of trabecular bone in 7-month-old mice but a marginal increase in 22-month-old mice.

**Table 5:** Trabecular Bone-Formation Indices From Mice Subjected to Axial Tibial Compression (Mean ± SD)

	7 Months						22 Months					
		
	8 N		10 N		12 N		8 N		10 N		12 N	
						
Outcome	Control (*n* = 12)	Loaded (*n* = 11)	Control (*n* = 12)	Loaded (*n* = 13)	Control (*n* = 8)	Loaded (*n* = 7)	Control (*n* = 11)	Loaded (*n* = 9)	Control (*n* = 8)	Loaded (*n* = 8)	Control (*n* = 7)	Loaded (*n* = 7)
sLS/BS (%)	23.7 ± 6.1	24.0 ± 6.2	21.7 ± 10.4	28.4^a^ ± 8.4	21.8 ± 10	29.9 ± 9.9	15.6 ± 10.3	19.7 ± 11.6	16.6 ± 7.1	25.6 ± 14.6	27.1 ± 9.8	33.2^b^ ± 12.2
dLS/BS (%)	1.6 ± 2.8	1.1 ± 1.8	0.7 ± 1.4	1.7 ± 3.5	0.2 ± 0.7	0.5 ± 1.4	0.0 ± 0.0	1.7 ± 2.9	0.0 ± 0.0	8.9 ± 21.8	0.4 ± 0.11	5.6 ± 8.4
MS/BS (%)	13.5 ± 4.2	13.1 ± 3.4	11.5 ± 5.2	15.8^a^ ± 4.4	11.1 ± 4.8	15.5^a^ ± 4.8	7.8 ± 5.1	11.6 ± 6.5	8.3 ± 3.6	21.7 ± 23.6	14.0 ± 5.6	22.2 ± 13.4
MAR (µm/day)	1.9 ± 0.6 (*n* = 4)	1.7 ± 0.1 (*n* = 4)	1.4 ± 0.5 (*n* = 4)	1.4 ± 0.4 (*n* = 5)	1.6(*n* = 1)	1.9 (*n* = 1)	ND	2.1 ± 1.5 (*n* = 3)	ND	2.1 ± 0.3 (*n* = 3)	1.5 (*n* = 1)	1.9 ± 0.05 (*n* = 3)
BFR/BS (µm/day)	0.28 ± 0.08 (*n* = 4)	0.24 ± 0.07 (*n* = 4)	0.15 ± 0.05 (*n* = 4)	0.22 ± 0.09 (*n* = 5)	0.14 (*n* = 1)	0.30 (*n* = 1)	ND	0.34 ± 0.33 (*n* = 3)	ND	0.96 ± 0.74 (*n* = 3)	0.34 (*n* = 1)	0.64 ± 0.20 (*n* = 3)

a Loaded (right) different from control (nonloaded, left); *p* < .05, paired *t* test.

b Different from 8-N group of same age; *p* < .05, ANOVA.

ND = no detectable double label; owing to the low incidence of double labels, statistical comparisons of MAR and BFR between loaded and nonloaded bones were not performed.

### Study 2: Effects of loading on trabecular bone

**Fig. 5:**
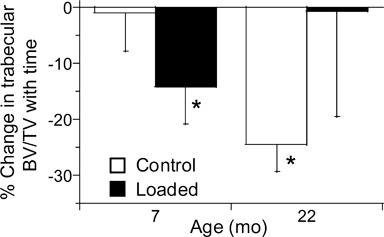
Percent change in trabecular BV/TV of the proximal tibial metaphysis based on in vivo µCT scans before and after 5 days of tibial compression (study 2) (mean ± SE). Loading caused trabecular bone loss in 7-month-old mice but appeared to prevent bone loss in 22-month-old mice. *After different from before; *p* < .05, paired *t* test.

Based on the observed differences in trabecular bone structure in loaded versus nonloaded tibias from study 1, we conducted a follow-up experiment using in vivo µCT to determine if there was trabecular bone loss with time. As in study 1, the effect of loading differed between age groups. In 7-month-old mice, trabecular BV/TV decreased significantly with time in loaded (*p* = .048) but not in control tibias (*p* = .82), indicating that loading caused loss of trabecular bone (Fig. 5; Table 6). Similar trends were noted in Tb.Th (*p* = .051) and vBMD (*p* = .09). In contrast, in 22-month-old mice, trabecular BV/TV did not change with time in loaded tibias (*p* = .88) but decreased significantly with time in control tibias (*p* = .002), suggesting that loading prevented bone loss in this age group. Finally, histomorphometric analysis of TRACP-labeled osteoclasts did not reveal differences in osteoclast surface between loaded and control tibias (Table 6).

**Table 6:** Trabecular (Tb) Bone Morphology and Density at the Proximal Tibial Metaphysis and Cortical (Ct) Bone Volume at the Mid-diaphysis Determined by µCT Before and After 5 Days of Tibial Loading—Osteoclast Surface Determined by Histology After Loading (Mean ± SD)

	7 Months				22 Months			
		
	Control (left)		Loaded (right)		Control (left)		Loaded (right)	
				
Outcome	Before (*n* = 12)	After (*n* = 12)	Before (*n* = 12)	After (*n* = 12)	Before (*n* = 12)	After (*n* = 7)	Before (*n* = 12)	After (*n* = 7)
Tb.BV/TV (mm^3^/mm^3^)	0.181 ± 0.20	0.178 ± 0.04	0.157^a^ ± 0.031	0.132^a^,^b^ ± 0.031	0.098 ± 0.031	0.075^b^ ± 0.017	0.085 ± 0.025	0.083 ± 0.046
Tb.N (1/mm)	4.4 ± 0.2	4.5 ± 0.3	4.5 ± 0.3	4.3 ± 0.3	3.9 ± 0.2	3.9 ± 0.2	3.8 ± 0.3	3.7 ± 0.3
Tb.Th (mm)	0.078 ± 0.004	0.077 ± 0.007	0.074^a^ ± 0.004	0.070^a^ ± 0.006	0.066 ± 0.008	0.060^b^ ± 0.005	0.064 ± 0.008	0.059 ± 0.005
Tb.vBMD (mg HA/cm^3^)	180 ± 15	175 ± 21	160^a^ ± 19	148^a^ ± 19	119 ± 24	109^b^ ± 15	108 ± 19	105 ± 29
Tb.Oc.S/BS (%)	—	2.8 ± 2.1	—	3.4 ± 2.4	—	3.0 ± 3.4	—	2.1 ± 1.0
Cort.BV (mm^3^)	0.378 ± 0.021	0.378 ± 0.025	0.385 ± 0.019	0.421^a^,^b^ ± 0.039	0.360 ± 0.029	0.345^b^ ± 0.027	0.359 ± 0.018	0.359 ± 0.017

a Loaded (right) different from control (nonloaded, left) at same time point.

b After different from before; *p* < .05, paired *t* test.

## Discussion

We subjected aged (22 months) and young-adult (7 months) mice to daily bouts of axial tibial compression for 1 week and evaluated their cortical and trabecular responses. We hypothesized that aged mice would be less responsive to loading than young-adult mice. At the tibial diaphysis, mice from both age groups showed a strong anabolic response, with a general finding of increasing bone formation with increasing load magnitude. Contrary to our hypothesis, at the endocortical surface, aged mice had a significantly greater response to loading than young-adult mice. Responses at the periosteal surface did not differ between age groups. At the proximal metaphysis, loading caused a loss of trabecular bone in young-adult mice, whereas it appeared to prevent bone loss in aged mice. Taken together, our results indicate no loss of short-term mechanoresponsiveness from age 7 to 22 months in the tibias of male BALB/c mice.

Our findings are in contrast to results of three previous studies that also used direct loading but concluded that cortical bone in the aged skeleton is relatively unresponsive. Rubin and colleagues compared the responses of aged (3 years) versus young-adult (1 years) turkeys to axial compression of the surgically isolated ulna (peak strain magnitude 3000 µɛ, 300 cycles/day) and concluded that a loading stimulus “that is clearly osteogenic in the young-adult skeleton is hardly acknowledged in older bone tissue.”(12) Similarly, when Turner and colleagues compared the responses of aged (19 months) versus young-adult (9 months) rats to tibial four-point bending (peak strains 900 to 1700 µɛ endocortically, 1600 to 3100 µɛ periosteally, 36 cycles/day), they found that the relative bone-formation rate in the older rats was 16-fold less than in the younger rats.(13) Finally, Srinivasan and colleagues compared the responses of aged (22 months) and young-adult (5 months) mice to tibial cantilever bending using a low-magnitude, rest-inserted protocol (peak strain 850 µɛ endocortically, 1250 µɛ periosteally, 50 cycles/day) and reported a 60% lower periosteal bone-formation rate in loaded tibias from the aged mice.(14)

The reasons for the difference in our findings from those of prior studies are unclear. We do not believe that it can be attributed to loading history. The range of strain magnitudes we applied (approximately 900 to 1900 µɛ endocortically and approximately 1400 to 3100 µɛ periosteally) is comparable with that used by Turner and colleagues.(13) At the low end of this range, magnitudes approximate those produced during physiologic activities in rodents(16,28) and are comparable with the “low magnitude” protocol of Srinivasan and colleagues.(14) The magnitude at the high end of the range is probably superphysiologic for rodents but matches the magnitude used by Rubin and colleagues.(12) Regarding waveform, we used a rest-inserted protocol to increase the likelihood of stimulating a response at low strain magnitude.(14,29) In our 8-N group, we did not observe an endocortical response but did detect a significant increase in periosteal MS/BS, similar to the results in the “low magnitude” group of Srinivasan and colleagues.(14) It is likely that differences in study design contributed to the difference in our results compared with others. First, we used an inbred mouse strain (BALB/c) different from the one used in the prior study of aged mice (C57Bl/6).(14) Different inbred strains have different mechanoresponsiveness,(30) and the effect of age on mechanoresponsiveness also may be strain-dependent. Among inbred strains, BALB/c mice have an intermediate bone mass and cortical thickness, whereas C57Bl/6 mice have low bone mass and thin cortices.(18,19,31) Nonetheless, both strains exhibit age-related changes that mimic human aging.(3,19) Another notable difference in our approach compared with the prior studies is that we used a noninvasive loading method that applies force along a physiologic direction. Perhaps the aged skeleton loses its ability to respond to nonhabitual modes of loading (eg, cantilever bending(14)) while retaining its ability to respond to a habitual loading mode when the magnitude is sufficient. Additional studies are needed to test this notion.

Although our findings are at odds with previous direct loading studies, they are consistent with several exercise studies. Two studies found that aged rats were more responsive to weight-bearing exercise than young-adult rats.(10,11) In one, Leppanen and colleagues reported that 2-year-old rats subjected to 12 weeks of treadmill running had significant increases in bone mass and strength at the femoral neck, whereas 1-year-old rats did not.(11) Moreover, several studies have reported that aged and young rats respond similarly to running and jumping protocols, concluding that the skeletal benefits of exercise were not limited by age.(7–9) On the other hand, two other studies in older rodents found reduced responsiveness to treadmill running compared with younger animals.(5,6) Nonetheless, the “positive” results cited earlier, taken together with our results, support the concept that under certain circumstances, the aged skeleton responds favorably to mechanical loading.

Whereas the cortical response to tibial compression in our study was strongly anabolic, the trabecular response was not. Dynamic histomorphometry indicated that loading modestly enhanced some measures of trabecular bone formation, yet µCT revealed a loss of bone in 7-month-old mice and no gain in 22-month-old mice. Taken together, these results suggest an increase in trabecular bone resorption in the 7-month-old group, although we were unable to detect an increase in osteoclast surface. Our trabecular findings are at odds with some, but not all, previous reports using the tibial compression model. Two-month-old mice had increased bone in the tibial metaphysis after 2 or 6 weeks of loading.(17,32) and in one study, 5-month-old mice also had a loading-related increase.(33) Interestingly, de Souza and colleagues reported that the trabecular response to 2 weeks of tibial compression was age-dependent, with increased trabecular BV/TV in 2-month-old old mice but decreased BV/TV in 3- and 5-month-old mice.(16) Our finding of trabecular bone loss after 1 week of tibial compression in young-adult mice is consistent the results of de Souza and colleagues. It remains unclear why this response was not also observed in the 22-month-old mice.

The trabecular results from our study and other studies using the tibial compression-loading model should be considered with the caveat that the loading environment in the trabecular bone is unknown. Whereas strains at the cortical mid-diaphysis can be measured with gauges, and simple equations then can be used to estimate peak endocortical and periosteal strains, the same approach cannot be applied to the metaphysis. Even if strain gauges were applied to the metaphyseal surface, they would not provide a measure of the local strains in the endosteal compartment. Finite-element analysis could be used to estimate trabecular strains, although this has not yet been reported. Thus, while cortical results between groups and between studies can be compared in the context of applied strain, the same does not hold for trabecular results. For this reason, our primary focus has been on the cortical response, and it is the cortical results that were used to test our hypothesis.

We chose 7 and 22 months as examples of young-adult and aged mice. Mice have an average lifespan on the order of 24 months. Their period of rapid skeletal growth ends by 4 months of age, and cortical bone mass is maintained through 12 months.(3,18,34,35) Consistent with prior reports,(3,4) we observed low indices of bone formation in control tibias from 7-month-old mice, indicating that by this age most bone surfaces are quiescent. The morphologic differences we observed between 7 and 22 months included endocortical expansion, cortical thinning, and loss of trabecular bone, consistent with age-related changes in mice reported by others.(3,31,35) These changes also mimic those which occur with aging in humans and support the clinical relevance of the aged mouse model.

One strength of our study was the use of in vivo µCT to assess temporal changes in trabecular bone. Typically, responses in rodent models of unilateral loading are determined by comparison of loaded versus control tibias, as we did in study 1. The loaded versus control comparisons of study 1 indicated less trabecular bone in loaded tibias (7-month-old group). This is consistent with the temporal decrease in trabecular BV/TV in loaded tibias (together with no change in control tibias) that we observed in study 2. Thus, in the 7-month-old groups the use of serial data was informative but did not lead to a different conclusion. In contrast, the use of serial scanning in 22-month-old mice provided insight not possible from postmortem scans. Trabecular BV/TV was not different between loaded and control tibias at the end of loading (studies 1 and 2). Yet serial scans revealed that there was a decrease in BV/TV in control but not in loaded tibias from 22-month-old mice, leading to a conclusion that loading appeared to prevent bone loss in this age group.

Several limitations should be considered when interpreting our findings. First, the strain magnitude produced by a given force tended to be less in the 22-month-old group than in the 7-month-old group. This is consistent with the trend for a greater moment of inertia in the older mice. However, because the strains values were not significantly different between groups, we chose not to adjust the force levels between groups. Had we increased the force level on the aged mice to produce a higher strain, it is likely we would have enhanced their bone-formation response, lending even further support to the conclusion that aged mice responded to loading. Second, we did not perform individualized strain analysis on each mouse that was subjected to in vivo loading. Thus the actual strains generated in these animals may have differed slightly from the estimates based on a priori analysis of a different set of mice. This may have contributed to variability in our results. Third, the force levels applied to the 7- and 22-month-old groups in study 2 were different. Because the trabecular results from study 1 were not affected by force level, it is unlikely that the difference in force level in study 2 had a significant effect. Lastly, we examined a 5-day loading period, which is relatively short compared with other studies of direct loading.(12–14,16,17) It is possible that a longer loading protocol would produce a different conclusion if, for example, the aged mice are not able to sustain their short-term response.

In summary, 1 week of daily tibial compression stimulated a robust endocortical and periosteal bone-formation response at the mid-diaphysis in both aged and young-adult male BALB/c mice. At the proximal metaphysis, loading appeared to maintain trabecular bone in aged mice while causing loss of trabecular bone in young adults. We conclude that aging does not limit the short-term anabolic response of cortical bone to mechanical stimulation in our animal model.
